# Response of iron overload to deferasirox in rare transfusion-dependent anaemias: equivalent effects on serum ferritin and labile plasma iron for haemolytic or production anaemias

**DOI:** 10.1111/j.1600-0609.2011.01660.x

**Published:** 2011-10

**Authors:** John B Porter, Kai-Hsin Lin, Photis Beris, Gian Luca Forni, Ali Taher, Dany Habr, Gabor Domokos, Bernard Roubert, Swee Lay Thein, on behalf of the EPIC study investigators

**Affiliations:** 1 University College LondonLondon, UK; 2 National Taiwan University HospitalTaipei, Taiwan; 3 Geneva University HospitalGeneva, Switzerland; 4 Ospedale GallieraGenoa, Italy; 5 American University of BeirutBeirut, Lebanon; 6 Novartis PharmaceuticalsEast Hanover, NJ, USA; 7 Novartis Pharma AGBasel, Switzerland; 8 Kings College HospitalLondon, UK

**Keywords:** rare anaemias, iron overload, iron chelation therapy, serum ferritin, safety

## Abstract

**Objectives:**

It is widely assumed that, at matched transfusional iron-loading rates, responses to chelation therapy are similar, irrespective of the underlying condition. However, data are limited for rare transfusion-dependent anaemias, and it remains to be elucidated if response differs, depending on whether the anaemia has a primary haemolytic or production mechanism.

**Methods:**

The efficacy and safety of deferasirox (Exjade®) in rare transfusion-dependent anaemias were evaluated over 1 yr, with change in serum ferritin as the primary efficacy endpoint. Initial deferasirox doses were 10–30 mg/kg/d, depending on transfusion requirements; 34 patients had production anaemias, and 23 had haemolytic anaemias.

**Results:**

Patients with production anaemias or haemolytic anaemias had comparable transfusional iron-loading rates (0.31 vs. 0.30 mL red blood cells/kg/d), mean deferasirox dosing (19.3 vs. 19.0 mg/kg/d) and baseline median serum ferritin (2926 vs. 2682 ng/mL). Baseline labile plasma iron (LPI) levels correlated significantly with the transfusional iron-loading rates and with serum ferritin levels in both cohorts. Reductions in median serum ferritin levels were initially faster in the production than the haemolytic anaemias, but at 1 yr, similar significant reductions of 940 and 617 ng/mL were attained, respectively (−26.0% overall). Mean LPI decreased significantly in patients with production (*P* < 0.0001) and haemolytic (*P* = 0.037) anaemias after the first dose and was maintained at normal mean levels (<0.4 μm) subsequently. The most common drug-related, investigator-assessed adverse events were diarrhoea (*n* = 16) and nausea (*n* = 12).

**Conclusions:**

At matched transfusional iron-loading rates, the responses of rare transfusion-dependent anaemias to deferasirox are similar at 1 yr, irrespective of the underlying pathogenic mechanism.

Regular red blood cell (RBC) transfusions are the principal supportive therapy for many rare anaemias. Although transfusion requirements may vary according to diagnosis, chronic transfusion therapy inevitably leads to iron overload that can cause significant damage to the heart, liver and endocrine glands (1, 2). In regularly transfused patients with β-thalassaemia, there are substantial data reporting the efficacy and safety of iron chelation therapy to treat iron overload (3–5). Data supporting the use of iron chelation therapy in other transfusion-dependent anaemias such as myelodysplastic syndromes (MDS), aplastic anaemia (AA) and sickle cell disease (SCD) are also accumulating (4–8) and suggest that response with respect to iron balance is mainly dependent on chelator dose and transfusional iron-loading rate (4, 9). Studies in patients with rare anaemias related to decreased RBC production or ‘production anaemias’ [including Diamond-Blackfan anaemia (DBA) and pure red cell aplasia] as well as those in patients with haemolytic anaemias have been limited (4), and response has not been analysed with respect to the underlying mechanism of anaemia. The 1-yr Evaluation of Patients’ Iron Chelation with Exjade® (EPIC) study enrolled a large number of patients with a variety of transfusion-dependent anaemias (5), thereby allowing the investigation of disease-specific considerations that might affect iron chelation therapy with deferasirox (Exjade®; Novartis Pharma AG, Basel, Switzerland). Data from patients with rare transfusion-dependent anaemias recruited into the EPIC study were included in these analyses.

The question as to whether the response to iron chelation therapy differs if the anaemia results primarily from decreased red cell production vs. when there is an underlying haemolytic mechanism remains to be answered. The rationale for the potentially variable response is that iron pools available for chelation may differ in haemolytic anaemias from those of production anaemias as a result of a greater rate of RBC catabolism. One approach to interrogating the access of chelators to iron pools is by the measurement of labile plasma iron (LPI); a directly chelatable redox-active subfraction of plasma non-transferrin bound iron (NTBI) (10). Cellular uptake of NTBI, through mechanisms including calcium channels (11) and zinc transporters (12), contributes to the iron-mediated cell damage (10, 13). NTBI and LPI are known to increase not only as a result of iron overload (14, 15) but also from ineffective erythropoiesis (16, 17) and when erythropoiesis is abrogated such as following myeloablation (18). LPI levels and the response to iron chelation may thus differ, depending on the underlying pathological mechanisms of anaemia. Therefore, in addition to assessing response of serum ferritin levels to deferasirox, LPI levels have been measured before and during iron chelation therapy. LPI levels have been compared between patients with haemolytic and production anaemias and related to the rates of transfusional iron loading and to serum ferritin levels.

## Methods

EPIC was a prospective, 1-yr, multicenter, open-label study that enrolled 1744 iron-overloaded patients with various transfusion-dependent anaemias, conducted across 23 countries. A full description of the study design and overall inclusion/exclusion criteria has been published previously (5).

### Key inclusion and exclusion criteria

Patients with rare transfusion-dependent anaemias from the EPIC study included in this study were male or female aged ≥2 yrs and with iron overload defined as serum ferritin levels of ≥1000 or <1000 ng/mL with a history of multiple transfusions (>20 transfusions or 1000 mL/kg of RBCs) and R2 magnetic resonance imaging-confirmed liver iron concentration (LIC) ≥2 mg Fe/g dry weight. These anaemias were classified as production or haemolytic in aetiology. Production anaemias were those where decreased red cell production was the underlying mechanism and included DBA and pure red cell aplasia. Patients with AA (19), MDS (8), thalassaemia or SCD (5) from the EPIC study were excluded from this analysis as they have been reported elsewhere. A definition of haemolysis was not included in the EPIC study protocol, and in this analysis, investigators have attributed haemolysis as the underlying mechanism on the basis of either a clear diagnosis [e.g. pyruvate kinase (PK) deficiency or autoimmune haemolytic anaemia (AIHA)] or the presence of one or more of the following features of haemolysis: raised reticulocyte count, raised lactate dehydrogenase (LDH), raised bilirubin, deleted plasma haptoglobin in addition to the absence of haemorrhage or production aetiology for the anaemia. Patients (or parents/guardians) provided written informed consent before entering the study, which was conducted in accordance with Good Clinical Practice guidelines and the Declaration of Helsinki. The study was approved by an Institutional Review Board/Independent Ethics Committee/Research Ethics Board at each participating site.

### Deferasirox dosing

Deferasirox starting dose was individualised, based on the frequency of blood transfusions; a starting dose of 20 mg/kg/d was recommended for patients receiving 2–4 RBC units/month (7–14 mL/kg/month), 30 mg/kg/d for patients receiving more frequent transfusions and 10 mg/kg/d for patients receiving less frequent transfusions. Protocol-specified dose adjustments were made in steps of 5–10 mg/kg/d (range 0–40 mg/kg/d), based on 3-monthly trends in serum ferritin and safety markers [including serum creatinine, transaminases and adverse events (AEs)]. Dose increases were recommended in patients with baseline serum ferritin values of >500 ng/mL who had an upward trend, or in patients with baseline serum ferritin values of >1000 ng/mL who had no downward trend after 3 months. Dose escalation above 40 mg/kg/d was permitted in exceptional circumstances and had to be approved individually by the Study Monitoring Committee. If serum ferritin levels fell to ≤500 ng/mL on two consecutive study visits, deferasirox treatment was suspended until levels increased to >500 ng/mL once more.

### Assessments

Serum ferritin levels were assessed every 4 wks. The primary efficacy endpoint was the change in serum ferritin levels from baseline to 1 yr. Secondary efficacy endpoints included evaluation of the relationship between dose adjustment regimens and transfusional iron overload. Blood samples to assess changes in levels of LPI using methods described previously (10) were taken preadministration (i.e. at predicted daily peak) and 2 h postdeferasirox administration, at baseline and Weeks 12, 28 and 52. Safety was evaluated throughout the study by regular monitoring and recording of AEs, routine laboratory testing and physical examination.

### Statistical methods

The efficacy population included all screened patients who had started deferasirox treatment; analysis was based upon the intent-to-treat principle. If no serum ferritin value was available at 1 yr, the last available observation was used as an end-of-study assessment to calculate the change from baseline (last-observation-carried-forward analysis). The safety population included all patients who had received at least one dose of study medication. Correlations were assessed using Pearson correlation coefficients. The reported *P*-values were based on two-sided paired tests at a 5% significance level (Student's *t*-test).

## Results

### Patient characteristics

Of the 57 patients with rare transfusion-dependent anaemias, 34 had anaemia resulting from decreased red cell production [production anaemias – pure red cell aplasia (*n* = 20) and DBA (*n* = 14)]. Twenty-three patients had anaemias resulting from a haemolytic process (haemolytic anaemias) including: haemolytic anaemia of uncharacterised cause (*n* = 11), pyruvate kinase deficiency anaemia (*n* = 5), autoimmune haemolytic anaemia (*n* = 4), congenital erythropoietic protoporphyria (*n* = 2), hereditary haemolytic anaemia (*n* = 1)]. Patient characteristics at baseline are shown in Table 1.

**Table 1:** Demographics and patient characteristics at baseline

	Production anaemias (*n* = 34)	Haemolytic anaemias (*n* = 23)	All patients (*n* = 57)
Mean age ± SD, years	35.2 ± 23.2	32.3 ± 22.6	34.0 ± 22.8
Age group, years, *n* (%)			
2–<16	8 (23.5)	6 (26.1)	14 (24.6)
≥16	26 (76.5)	17 (73.9)	43 (75.4)
Male:female, *n*	15:19	12:11	27:30
Race (Caucasian:Oriental:other), *n*	28:4:2	14:8:1	42:12:3
History of hepatitis B or C, *n* (%)	3 (8.8)	2 (8.7)	5 (8.8)
History of splenectomy, *n* (%)	1 (2.9)	11 (47.8)	12 (21.1)
Previous iron chelation therapy, *n* (%)			
DFO monotherapy	21 (61.8)	13 (56.5)	34 (59.6)
Deferiprone monotherapy	1 (2.9)	–	1 (1.8)
DFO and deferiprone^1^	5 (14.7)	2 (8.7)	7 (12.3)
Chelation-naïve	7 (20.6)	8 (34.8)	15 (26.3)
Mean duration of previous iron chelation therapy ± SD, years	6.2 ± 8.6 (*n* = 27)	5.2 ± 7.5 (*n* = 15)	5.8 ± 8.2 (*n* = 42)
Mean duration of transfusion history ± SD, years	9.9 ± 10.2	13.8 ± 12.7	11.5 ± 11.4
Mean number of transfusion sessions in the year prior to study entry ± SD	21.5 ± 18.1	19.0 ± 19.5	20.5 ± 18.5
Mean volume transfused in the year prior to study entry ± SD, mL/kg	152 ± 118	174 ± 174	161 ± 142

DFO, deferoxamine; SD, standard deviation.

1 patients received both DFO and deferiprone as prior chelation therapies, but these may not have been in combination.

### Prior chelation history

Overall, more than two-thirds of patients had received prior chelation therapy for an approximate mean period of 6 yrs. Deferoxamine was the most frequently used therapy prior to study (Table 1). In previously chelated patients, the mean duration of previous iron chelation therapy was similar for production and haemolytic anaemias (Table 1).

### Deferasirox dosing and adjustments

Forty-eight patients (84.2%) started deferasirox at 20 mg/kg/d; 31 (91.2%) with production anaemias and 17 (73.9%) with haemolytic anaemias. The overall mean planned dose was not different in production anaemias (20.3 ± 3.0 mg/kg/d) compared with haemolytic anaemias (17.6 ± 4.2 mg/kg/d), neither was the median time to dose increment (20 wks, range 4–48 and 20 wks, range 9–48, respectively). Final daily doses of 10, 20, 25, 30 and 40 mg/kg/d occurred in four, 10, five, five and one patient in the production group and seven, seven, zero, four and one patient in the haemolytic group. The number of patients requiring dose reductions because of laboratory abnormalities was higher among patients with production anaemia (*n* = 9, 26.5%) compared with patients with haemolytic anaemia (*n* = 2, 8.7%). Similarly, dose interruptions for AEs were more common in the production (*n* = 14, 41.2%) than the haemolytic cohorts (*n* = 6, 26.1%). The mean actual dose over 1 yr was similar in the production (19.3 ± 5.3 mg/kg/d) and haemolytic (19.0 ± 5.7 mg/kg/d) cohorts.

### Transfusional iron loading

The mean durations of previous transfusion history were similar for the production anaemias and haemolytic anaemias (Table 1). The mean transfusion requirements in the year prior to the study were also similar (Table 1) with comparable iron-loading rates during the study (Table 2). This is also similar to mean rates reported for MDS and thalassaemia major in the EPIC study (5). The rates and patient numbers differ from rare anaemias reported in the previous EPIC paper (5) because some patients previously classified as ‘other’ have been included in the current analysis of rare anaemias.

**Table 2:** Transfusional iron-loading rate and response to deferasirox

	Production anaemias (*n* = 34)	Haemolytic anaemias (*n* = 23)	All patients (*n* = 57)
Mean ± SD iron intake rate, mg/kg/d	0.34 ± 0.14 (*n* = 30)	0.33 ± 0.24 (*n* = 20)	0.33 ± 0.18 (*n* = 50)
Median baseline serum ferritin (range), ng/mL	2926 (908–10861) (*n* = 33)	2682 (568–13078) (*n* = 22)	2916 (568–13078) (*n* = 56)
Median serum ferritin at end-of-study (range), ng/mL	1972 (145–10700) (*n* = 33)	1855 (454–9969) (*n* = 22)	1941 (145–10700) (*n* = 56)
Median absolute change in serum ferritin, ng/mL	−940 (*P* = 0.06) (*n* = 33)	−617 (*P* = 0.02) (*n* = 22)	−832 (*P* = 0.005) (*n* = 56)
Mean baseline LPI ± SD, μm	0.56 ± 0.5 (*n* = 22)	0.35 ± 0.5 (*n* = 14)	0.48 ± 0.5 (*n* = 36)
Mean LPI at end-of-study predose ± SD, μm	0.16 ± 0.3 (*n* = 18)	0.29 ± 0.6 (*n* = 17)	0.22 ± 0.5 (*n* = 35)
Mean LPI at end-of-study postdose ± SD, μm	0.01 ± 0.0 (*n* = 17)	0.01 ± 0.0 (*n* = 15)	0.01 ± 0.0 (*n* = 32)

RBC, red blood cells; SD, standard deviation; LPI, labile plasma iron.

### Effect of deferasirox on absolute and relative serum ferritin levels

**Figure 1:**
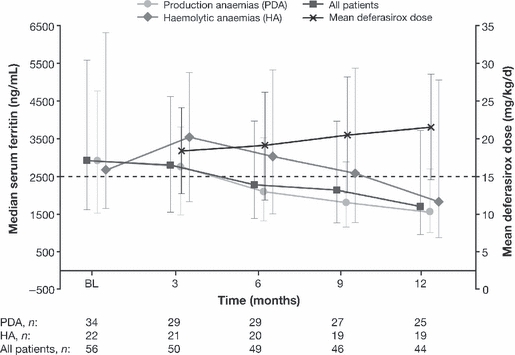
Median serum ferritin levels (±25th/75th percentiles) and mean deferasirox dose (±SD) for all patients over the 1-yr study. *Dotted line indicates a threshold of 2500 ng/mL, serum ferritin levels above which are associated with significant negative outcomes in thalassaemia major (20, 21).

Overall, baseline median serum ferritin levels were >2500 ng/mL (Table 2). After 1 yr of treatment with deferasirox, median serum ferritin levels were reduced to <2000 ng/mL in both patient cohorts (Fig. 1 and Table 2 respectively), and 12 patients were able to achieve serum ferritin levels of <1000 ng/mL; five patients with haemolytic anaemias, four with DBA and three with pure red cell aplasia. The percentage reduction in serum ferritin was similar in production (27.1%) and haemolytic anaemias (24.1%). The decrease in median serum ferritin levels was significant in patients with haemolytic anaemias (*P* = 0.02 based on last-observation-carried-forward analysis) (Table 2). However, in patients with production anaemias, despite a clinically relevant median absolute reduction in ferritin levels, this did not reach statistical significance (*P* = 0.06). This may have been attributed to one patient outlier with pure red cell aplasia who had an absolute increase in serum ferritin levels of >7000 ng/mL from baseline to end-of-study associated with rapid weight loss (5.2 kg over 1 month from treatment day 338–366) and high levels of alanine aminotransferase (ALT) and aspartate aminotransferase (585 and 390 U/L, respectively at treatment day 366), which may have been indicative of acute hepatitis. When omitted from the analysis, median change in serum ferritin for patients with production anaemias was significant (*P* = 0.0009). Subcategorisation of patients with DBA also showed a significant reduction in median serum ferritin levels from a median baseline of 2289 to 1521 ng/mL at end-of-study (median absolute reduction of 790 ng/mL; *P* = 0.0121). It should be noted from Fig. 1 that patients with haemolytic anaemia experienced a transient increase in serum ferritin levels at 3 months, which subsequently decreased resulting in comparable serum ferritin values to the production anaemia at 1 yr.

### Effect of deferasirox on labile plasma iron levels

**Figure 2:**
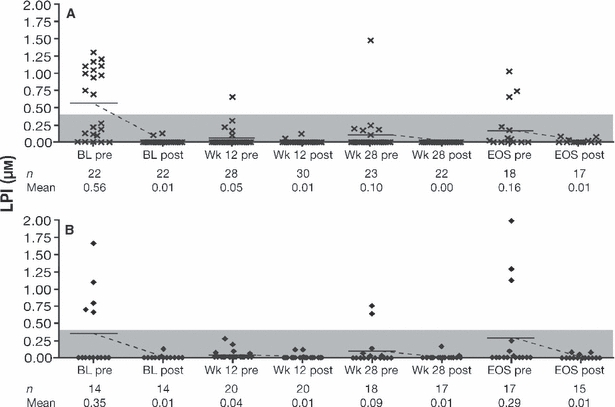
Labile plasma iron (LPI) levels, pre- and postdose administration at baseline and after repeat doses. Individual patient data are shown for patients with (A) production anaemias and (B) haemolytic anaemias. The grey-shaded area indicates a LPI threshold of 0.4 μm (22). Horizontal bars denote mean LPI.

Mean predose LPI levels are shown in Table 2. Values above normal reference limits (>0.4 μm) (22) were found in 11 of 22 patients with production anaemias and in five of 14 patients with haemolytic anaemias (Fig. 2). Overall, mean predose LPI was not significantly different in chelation-naïve versus previously chelated patients in both production (*P* = 0.306) and haemolytic anaemias (*P* = 0.809). LPI levels decreased from predose values for both cohorts, with normalisation (<0.4 μm) of the mean for all patients at all points in the study (Fig. 2). For patients with production anaemias, the reduction in predose LPI levels was significant after the first dose (*P* < 0.0001), and at Weeks 12 (*P* = 0.0002), 28 (*P* = 0.006) and 52 (end-of-study) (*P* = 0.014) compared with baseline. When excluding the patient with a reported absolute increase from baseline in serum ferritin level to >7000 ng/mL, the results remained significant (*P* < 0.0001, *P* = 0.0005, *P* = 0.012 and *P* = 0.029, respectively). A ‘rebound’ effect was noted in some patients at Week 52 (end-of-study) predose assessments. Of the patients with production anaemias with predose LPI levels >0.4 μm at Week 52 (end-of-study) (*n* = 3), all had pure red cell aplasia; one patient had an increase in serum ferritin level >7000 ng/mL from baseline at the end-of-study as previously discussed; one patient achieved a reduction in serum ferritin levels of 1709 ng/mL at end-of-study compared with baseline, despite having a higher than average transfusion rate (0.53 mL RBC/kg/d); and the third patient achieved a reduction in serum ferritin level of 1658 ng/mL and had a lower than average transfusion rate (0.33 mL RBC/kg/d). Mean deferasirox dose at end-of-study in these three patients was 29.8, 10.6 and 28.4 mg/kg/d, respectively. All three patients completed the study.

For patients with haemolytic anaemias, the reductions in predose LPI levels were significant after the first dose (*P* = 0.037) and at Week 12 (*P* = 0.048) compared with baseline. As with production anaemias, some patients with haemolytic anaemia (*n* = 3) demonstrated a recurrence of LPI predose at Week 52 (end-of-study) with predose LPI levels >0.4 μm; these had underlying diagnoses of hereditary haemolytic anaemia, acute porphyria and pyruvate kinase deficiency, respectively. The patient with hereditary haemolytic anaemia achieved a reduction in serum ferritin levels of 771 ng/mL but discontinued the study because of withdrawal of consent. The remaining two patients with end-of-study LPI values >0.4 μm completed the study, and both had increases in serum ferritin levels compared with baseline (+1254 and +1079 ng/mL, respectively) with transfusion rates lower than average (0.23 and 0.18 mL RBC/kg/d, respectively). Mean deferasirox dose at end-of-study in these three patients was 25.1, 18.9 and 28.4 mg/kg/d, respectively.

### Relationship of baseline LPI to iron-loading rate and serum ferritin

**Figure 3:**
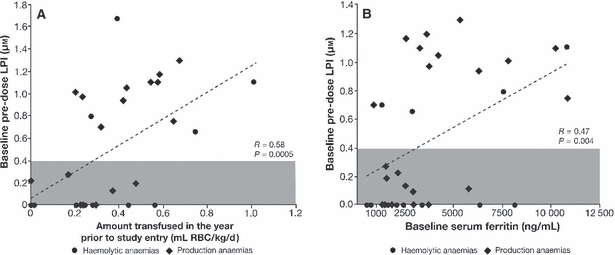
(A) The relationship between baseline predose labile plasma iron (LPI) and transfusion rate in the year prior to study entry. There is a significant correlation (*R* = 0.58, *P* = 0.0005, *n* = 32) between transfusion rate in the year prior to study entry and baseline predose LPI in all patients. Haemolytic anaemias are shown in circles and production anaemias in diamonds. The grey area denotes the healthy reference range. (B) The relationship between baseline predose LPI and baseline serum ferritin. There is a significant relationship (*R* = 0.47, P = 0.004) between baseline ferritin and baseline predose LPI for all patients. Haemolytic anaemias are shown in circles and production anaemias in diamonds. The grey area denotes the healthy reference range.

Analysis of available predose LPI measurements at baseline in all patients indicated a significant correlation with the rate of transfusion (mL RBC/kg/d) in the year prior to study entry [*R =* 0.58, *P* = 0.0005 (*n* = 32); Fig. 3A]. Patients with haemolytic (circles) and production (diamonds) anaemias appear to fall broadly within this same relationship. Predose LPI measurements at baseline were also correlated with transfusional iron-loading rate (mg/kg/d) over the study (*R =* 0.56, *P* = 0.001; not shown). Transfusional iron-loading rate (mg/kg/d) over the study was correlated with LPI levels at 12 wks (*R =* 0.39, *P* = 0.01; not shown), but by 28 wks (*R =* 0.21, *P* = 0.22; not shown) and end-of-study (*R =* 0.06, *P* = 0.76; not shown), this relationship was not apparent. This is likely to reflect the progressive and effective suppression of LPI by deferasirox so that the contribution of the iron-loading rate to LPI levels progressively decreased. Abnormal predose LPI values at baseline were found only in patients with transfusional iron-loading rates >0.2 mg/kg/d.

Pretreatment LPI also shows a significant correlation with baseline serum ferritin levels [*R =* 0.47, *P* = 0.0043 (*n* = 35); Fig. 3B]. Patients with haemolytic (circles) and production (diamonds) anaemias appear to fall broadly within this same relationship. This relationship of predosing LPI and serum ferritin also correlated significantly at end-of-study (*R =* 0.47, *P* = 0.004; not shown). However, unlike the relationship to transfusional loading rate, there was no serum ferritin value below which abnormal LPI values were absent (Fig. 3B).

### Tolerability of deferasirox over 1 yr

Overall, 44/57 (77.2%) patients completed 1 yr of treatment. In patients with production anaemias, six discontinued (all with pure red cell aplasia) because of AEs (increased serum creatinine, oedema, abdominal pain, chronic renal failure, hyperpyrexia and dyspepsia), two died (one as a result of cardiac and respiratory failure and one as a result of bladder neoplasm, neither of which were considered related to study drug) and one discontinued because of a protocol violation. Two patients from the cohort with haemolytic anaemias discontinued because of an AE (angioedema and cholelithiasis, respectively), one patient withdrew consent and one patient discontinued because of unsatisfactory therapeutic effect. The most common drug-related AEs as assessed by investigators were gastrointestinal: diarrhoea (28.1%), nausea (21.1%), abdominal pain (12.3%), upper abdominal pain (10.5%), vomiting (7.0%), abdominal distension (5.3%) and constipation (5.3%). Other AEs assessed by the investigators were as follows: increased serum creatinine (12.3%), rash (10.5%), ALT increases (8.8%), fatigue (8.8%), pruritus (5.3%) and dizziness (5.3%). There were no serious AEs considered by the investigators to be drug-related in either patient cohort.

When strict criteria of a > 33% creatinine increase and a value greater than upper limit of normal on two consecutive occasions were used, eight patients had such an increase, three with production anaemias and five with haemolytic anaemias. For the patients with haemolytic anaemias, three received dosing reductions as a result, from 20 to 10 mg/kg/d (*n* = 2) and from 30 to 20 mg/kg/d (*n* = 1). Serum creatinine levels then decreased and these patients completed the study. The other two patients completed the study without any dose alterations. Of the three patients with production anaemias (all with pure red cell aplasia), one continued the study without any dose alterations (although serum creatinine levels remained high but subsequently discontinued because of dyspepsia). For the other two patients, the deferasirox dose was initially reduced from 20 to 10 mg/kg/d. Following dose reduction, serum creatinine levels decreased in one of these, but remained high compared with baseline levels, resulting in eventual discontinuation from the study.

In total, four patients received cyclosporine. Two patients completed the study without drug discontinuation; one (described above) received a dose reduction because of serum creatinine levels (>33% above baseline and greater than upper limit of normal on two consecutive visits); the other patient had a stable serum creatinine level throughout the study (baseline serum creatinine of 57 μm; range throughout the study 55–65 μm). Two of the four patients receiving concomitant cyclosporine discontinued, one discontinued because of chronic renal failure having experienced elevated creatinine (105 and 146 μm on study days 1 and 196, respectively). This patient also experienced multiple AEs during the study including pancytopenia, bronchitis, postallograft lymphoproliferative syndrome and persistent fever that were not considered to be related to chelation therapy by the study investigator. The second patient discontinued because of cardiac/respiratory failure.

ALT levels >10× upper limit of normal on two consecutive visits were seen in two patients with DBA. These patients also had elevated ALT levels at baseline (301 and 130 U/L) but had no reported history of hepatitis B/C. The investigators did not adjust dose and both patients completed the study, with end-of-study ALTs of 154 and 92 U/L. One patient with pure red cell aplasia, who also had a raised baseline ALT (254 U/L), developed ALT levels >10× upper limit of normal on two consecutive visits and subsequently discontinued because of abdominal pain.

## Discussion

This is the first study to examine how responses of rare transfusion-dependent anaemias to deferasirox chelation are affected by the underlying production or haemolytic mechanisms. Because of their rarity, such patients may not be monitored or treated as consistently as those with thalassaemia major (23). In this study, even though many patients had received prior chelation, in over half of the patients, baseline serum ferritin values were at levels typically associated with negative outcomes in thalassaemia major (20, 21). Reductions in serum ferritin levels were clinically relevant for both production and haemolytic anaemias, supporting a dosing approach tailored to baseline serum ferritin measurements with subsequent dose adjustment, based on serum ferritin trend analysis every 3 months. Overall, the mean transfusional iron-loading rates, doses of deferasirox received and final serum ferritin responses were similar in production and haemolytic anaemias, suggesting no fundamental difference in chelation response.

The delayed serum ferritin response in the haemolytic group relative to the production group is of interest. The mechanism is not clear but may relate to a higher proportion of iron being present in the reticuloendothelial compartment in haemolytic anaemias compared with production anaemias. Serum ferritin up to values of 4000 μg/L is predominantly derived from macrophages of the reticuloendothelial system (24). The higher rate of red cell catabolism within macrophages of haemolytic patients may result in continued high ferritin synthesis in this compartment, even when iron is being effectively chelated within hepatocytes. There is also some evidence using liver biopsies which suggests faster iron chelation by deferasirox from hepatocytes compared with macrophages in the initial stages of chelation therapy (Yves Deugnier, personal communication). Both of these effects would mean that serum ferritin decrements would tend to lag behind decrements in LIC, particularly with haemolytic anaemias. However, the similar serum ferritin values in the haemolytic and production anaemias at 1 yr suggest that macrophage iron is eventually accessed as effectively in haemolytic anaemias as in production anaemias.

In patients with DBA, the reduction in serum ferritin was comparable to other anaemias in the EPIC study, but in a previous Phase II study, serum ferritin decrements were less than in other patients (including patients with β-thalassaemia and MDS) (4). This was previously attributed to comparatively higher transfusional iron intake for DBA patients (4), but here, the iron-loading rates for DBA (0.37 ± 0.12 mg/kg/d) are similar to other rare anaemias and may explain why the serum ferritin response is also similar.

The measurement of LPI may give insights into the effectiveness and duration of action of chelation therapy (22), but its value in the routine monitoring of iron overload and chelation therapy remains unclear. Deferasirox produces sustained decrements in LPI in β-thalassaemia (25) and MDS (26–28), but this is the first time that LPI levels have been assessed in rare transfusion-dependent anaemias. The correlation of LPI with the transfusional rate has not been previously reported in any form of transfusional iron overload and suggests that the transfusional loading rate may be an important factor in determining plasma levels of iron species responsible for pathological iron distribution. Once deferasirox chelation is ongoing, LPI is largely determined by the plasma levels of chelator (22); thus, no correlation between LPI and iron intake rate was observed at later time points. Previous studies have provided indirect evidence linking chelatable iron (as assessed by urine iron with deferoxamine therapy) and myocardial iron (29). As LPI is a key chelatable form of plasma iron, the findings here suggest a link could also exist between transfusional loading rates and the risk of myocardial iron loading from LPI or other NTBI species. As myocardial T2* was not measured in this study, this merits systematic investigation.

The correlation of baseline LPI with serum ferritin is consistent with findings in untransfused β-thalassaemia/haemoglobin E (15) but has not been previously demonstrated in transfusion-dependent anaemias. Despite the relationship of LPI with serum ferritin, there was no clear ferritin ‘cut-off’ below which LPI was always in the normal range (Fig. 3B). Conversely, LPI was often within the normal range (<0.4 μm) even though ferritin values exceeded 1000 ng/mL (Fig. 3B). It is noteworthy that in a previous study of AA (19), LPI was not increased at baseline, despite similar mean iron-loading rates to the production anaemias described in this study. The reason is unclear and requires further investigation. As with thalassaemia major (25), there was an immediate postdosing decrease in LPI, but unlike thalassaemia major patients in the EPIC study (30), all rare transfusion-dependent anaemias had values in the normal range after the first dose. The predosing trough levels are clearly decreased at 12 wks, but at end-of-study assessments, there was a ‘rebound’ in some patients. As the removal of LPI by deferasirox correlates with drug concentrations (25), the measurement of trough levels in patients with unexplained late LPI resurgence may be helpful in establishing whether inadequate drug exposure, either from reduced compliance or drug absorption, is responsible.

Deferasirox was generally well tolerated with predominantly gastrointestinal and rash-related AEs, consistent with other anaemias such as β-thalassaemia and SCD (4, 7, 31) as well as the overall EPIC study (5). Renal and hepatic safety were managed using monitoring recommendations in line with the product label for deferasirox (4, 5, 32). Increments in serum creatinine levels >33% above baseline and greater than upper limit of normal were generally managed with dose decreases and/or interruptions and without subsequent progressive increments in plasma creatinine. More patients with production anaemias or, more specifically, those with pure red cell aplasia, discontinued the study as compared with haemolytic anaemias, and these patients also experienced a higher frequency of AEs, dose reductions and dose interruptions. The reasons are not obvious but may relate to previous treatments and/or drug exposure. For example, two patients with pure red cell aplasia, requiring discontinuation, were receiving concomitant cyclosporine, which may have contributed to the rise in serum creatinine (33). A rise in creatinine with deferasirox therapy, more than 33% above baseline and above upper limit of normal, was previously noted to be three times as likely in patients with AA also receiving concomitant cyclosporine (19). Careful monitoring is therefore advisable for patients receiving concomitant cyclosporine or other potentially nephrotoxic medications.

In conclusion, these data provide evidence that transfusional iron overload in patients with a variety of rare anaemias may be effectively managed using a tailored dosing deferasirox regimen, based on individual blood transfusion requirements, regular monitoring of serum ferritin trends and safety parameters. These data also indicate that, for patients with similar rates of transfusional iron loading, the trends in serum ferritin and LPI reduction following treatment with deferasirox are essentially the same, irrespective of the underlying condition requiring chronic blood transfusions.

## Appendix

### Participating centres and investigators

L. Agaoglu, Istanbul University, Medical Faculty, Capa, Istanbul, Turkey; G. Alimena, Az. Policlinico Umberto I, Roma, Italy; D. Alonso, Hospital Universitario Virgen del Rocio, Sevilla, Spain; S. Ame, Hôpital Hautepierre, Strasbourg, France; E. Angelucci, Ospedale Oncologico A. Businco, Cagliari, Italy; B. Arrizabalaga, Hospital de Cruzes, Barakaldo, Spain; M. Athanasiou-Metaxa, Aristotle University of Thessaloniki, Thessaloniki, Greece; B. Augustson, Sir Charles Gairdner Hospital, Perth, Australia; Y. Aydinok, Ege University, Medical Faculty, Bornova, Izmir, Turkey; A. Baba, Hospital University Sains Malaysia, Kota Bahru, Malaysia; M. Baccarani, Az. Ospedaliera di Bologna, Malpighi, Bologna, Italy; J. Beck, Klinikum der Universität Mainz, Mainz, Germany; O. Beyne-Rauzy, Hôpital Purpan, Toulouse, France; H. Birgens, Amtssygehuset i Herlev, Herlev, Denmark; D. Bordessoule, CHU Limoges, Limoges, France; C. Borgna-Pignatti, Az. Osp. Universitaria Sant'Anna, Ferrara, Italy; A. Bosly, Cliniques Universitaires U.C.L., Godinne, Belgium; K. Bouabdallah, CHU Bordeaux, Bordeaux, France; D. Bowden, Monash Medical Centre, Melbourne, Australia; D. Bowen, Leeds General Infirmary, Leeds, UK; D. Bron, Institut Jules Bordet, Brussels, Belgium; M. D. Cappellini, Universitá di Milano, Ca Granda Foundation IRCCS, Milan, Italy; M. Capra, Ospedale Civico G. di Cristina M. Ascoli, Palermo, Italy; G. Cartron, Clinique Victor Hugo, Le Mans, France; M. Cazzola, Policlinico S. Matteo IRCCS, Pavia, Italy; C. Chalkias, General Hospital of Larissa, Larissa, Greece; L. L. Chan, University Malaya Medical Centre, Kuala Lumpur, Malaysia; S. Chancharunee, Ramathibodi Hospital, Mahidol University, Bangkok, Thailand; C. Chapman, Leicester Royal Infirmary, Leicester, UK; P. Charoenkwan, Chiang Mai University, Chiang Mai, Thailand; E. Chasapopoulou, University Hospital of Thessaloniki AHEPA, Thessaloniki, Greece; S. Cheze, CHR Clemenceau, Caen, France; A. Chuansumrit, Ramathibodi Hospital, Mahidol University, Bangkok, Thailand; P. Cianciulli, Ospedale S. Eugenio, Roma, Italy; C. Dauriac, CHRU Rennes, Hôpital Pontchaillou, Rennes, France; M. Delforge, UZ Gasthuisberg, Leuven, Belgium; G. Dölken, Ernst-Moritz-Arndt-Universität Greifswald, Greifswald, Germany; H. Dombret, Hôpital Saint Louis, Paris, France; J. Duyster, Klinikum rechts der Isar der TU München, München, Germany; T. Economopoulos, Athens University Medical School, Xaidari, Greece; G. Ehninger, Universitätsklinikum Dresden, Dresden, Germany; M. Elalfy, Ain Shams University, Cairo, Egypt; A. El-Beshlawy, Cairo University, Cairo, Egypt; L. Enggaard, Hillerod Sygehus, Hillerod, Denmark; P. Fenaux, Hôpital Avicenne, Bobigny, France; G. Fillet, Centre Hospitalier Universitaire Sart Tilman, Liège, Belgium; A. Filosa, Az. Osp. A. Cardarelli, Napoli, Italy; R. Galanello, Ospedale Regionale Microcitemie, Cagliari, Italy; A. Ganser, Kliniken der Med. Hochschule Hannover, Hannover, Germany; G. Gastl, Uni-Klinik Innsbruck, Innsbruck, Austria; N. Gattermann, Heinrich-Heine-University, Düsseldorf, Germany; S. Giraudier, Hôpital Henri Mondor, Créteil, France; A. Goldfarb, Hadassah Medical Centre, Jerusalem, Israel; A. Grigg, Royal Melbourne Hospital, Melbourne, Australia; A. Guerci-Bresler, CHU Nancy, Vandoeuvre Les Nancy, France; F. Gumruk, Hacettepe University, Medical Faculty, Sihhiye, Ankara, Turkey; S. Y. Ha, Queen Mary Hospital, Hong Kong, Hong Kong; D. Haase, Universitätsklinikum Göttingen, Göttingen, Germany; B. Heinrich, Gemeinschaftspraxis Brudler/Heinrich, Ausburg, Germany; M. Hertzberg, Westmead Hospital, Sydney, Australia; J. Ho, Royal Prince Alfred Hospital, Sydney, Australia; H-C. Hsu, Taipei Veterans General Hospital, Taipei, Taiwan; S. Huang, Guang Zhou Zhong Shan No.2 Hospital, Guangzhou, China; M. Hunault-Berger, CHU d'Angers, Angers, France; B. Inusa, Evelina Childrens Hospital, London, UK; D. Jaulmes, Hôpital Saint Antoine-Paris, Paris, France; J. Jensen, Arhus Sygehus, Arhus, Denmark; A. Kattamis, First Department of Pediatrics, University of Athens, Athens, Greece; Y. Kilinc, Cukurova University, Medical Faculty, Balcali, Adana, Turkey; K-H. Kim, Samsung Medical Centre, Seoul, South Korea; S. Kinsey, St James’ University Hospital, Leeds, UK; L. Kjeldsen, Rigshospitalet, Copenhagen, Denmark; A. Koren, Ha'emek Medical Centre, Afula, Israel; M. E. Lai, Ospedale Regionale Microcitemie, Cagliari, Italy; Y. Lai, No.1 hospital of Nanjing Medical University, Nanjing, China; J-W. Lee, The Catholic University of Korea, Seoul, South Korea; K-H. Lee, Asan Medical Centre, Seoul, South Korea; S-H. Lee, Royal Adelaide Hospital, Adelaide, Australia; L. Legros, Hôpital de l'Archet, CHU de Nice, Nice, France; C. Li, Guang Zhou Nang Fang Hospital, Guangzhou, China; C-K. Li, Prince of Wales Hospital, Chinese University of Hong Kong, Hong Kong; Q. Li, Shanghai No.1 Hospital, Shanghai, China; W. Linkesch, Med. Univ. Klinik Graz, Graz, Austria; M. Lübbert, Universitätsklinikum, Freiburg, Germany; D. Lutz, A.Ö. Krankenhaus der Elisabethinen, Linz, Austria; A. J. Mohamed Thalha, Hospital Universiti Kebangsaan, Kuala Lumpur, Malaysia; G. Mufti, King's College Hospital, London, UK; P. Muus, UMC St. Radboud, Nijmegen, The Netherlands; F. Nobile, Az. Osp. Bianchi Melacrino Morelli, Reggio Calabria, Italy; H. Ozsahin, Children's Hospital, Geneva, Switzerland; N. Papadopoulos, Aristotle University of Thessaloniki, Thessaloniki, Greece; S. Perrotta, l Policlinico ll Università di Napoli, Napoli, Italy; M. Petrini, Az. Osp. Ospedali Riuniti S. Chiara, Pisa, Italy; M. Pfeilstöcker, Hanusch-Krankenhaus, Wien, Austria; A. Piga, Ospedale Regina Margherita, Torino, Italy; J. Poole, Johannesburg Hospital, Johannesburg, South Africa; E. Pungolino, Az, Osp. Niguarda Ca’ Granda – Università degli Studi di Milano, Milan, Italy; G. Quarta, Ospedale A. Perrino, Brindisi, Italy; C. Ravoet, H.H. Jolimont Lobbes (Jolimont), La Louviere, Belgium; A. F. Remacha, Hospital de la Santa Creu i San Pau, Barcelona, Spain; C. Rose, Hôpital Saint-Vincent de Paul (Groupe Francophone des Myélodysplasies), Lille, France; L. Roy, Centre Jean Bernard, Poitiers, France; G. Saglio, Az. Sanitaria Ospedale S. Luigi Gonzaga, Orbassano, Italy; G. Sanz, Hospital La Fe, Valencia, Spain; M. Schmid, Universität Ulm, Ulm, Germany; M. Schmugge, Kinderspital Zürich, Zürich, Switzerland; H. Schots, Universitair Ziekenhuis Gent, Gent, Belgium; G. Secchi, Ospedale Civile, Azienda USL 1, Sassari, Italy; J. F. Seymour, Peter MacCallum Cancer Centre, Melbourne, Australia; F. Shah, Whittington Hospital, London, UK; H. Shah, General Hospital Kuala Lumpur, Kuala Lumpur, Malaysia; Z. Shen, Shang Hai Rui Jin Hospital, Shanghai, China; B. Slama, Centre Hospitalier General, Avignon, France; P. Sutcharitchan, Chulalongkorn University, Bangkok, Thailand; H. Tamary, Schneider Children's Medical Centre, Petach Tikva, Israel; K. Taylor, Mater Hospital, Brisbane, Australia; H-J. Tesch, Onkologische Gemeinschaftspraxis, Frankfurt, Germany; J. Troncy, Hôpital Edouard Herriot, Lyon, France; D. Vassilieff, Hôpital Cochin, Paris, France; A. Villegas, Hospital Clinico Universitario San Carlos, Madrid, Spain; V. Viprakasit, Siriraj Hospital, Mahidol University, Prannok, Bangkoknoi, Bangkok, Thailand; L. Wainwright, Chris Hani Baragwanath Hospital, Johannesburg, South Africa; B. Wassmann, Johann Wolfgang Goethe Universität Frankfurt, Frankfurt, Germany; M. Wettervald, CHU Dunkerque, Dunkerque, France; A. Will, Manchester Children's Hospital, Pendlebury, UK; B. Wörmann, Städt. Klinikum Braunschweig, Braunschweig, Germany; J. Wright, Royal Hallamshire Hospital, Sheffield, UK; S-P. Yeh, China Medical University Hospital (Taichung), Taichung, Taiwan; S-S. Yoon, Seoul National University Hospital, Seoul, South Korea; N. C. Zoumbos, Patras University Medical School, Patras IRO, Greece; S. Zweegman, VUMC, Amsterdam, The Netherlands.
